# *In Vitro* Conservation of Twenty-Three Overexploited Medicinal Plants Belonging to the Indian Sub Continent

**DOI:** 10.1100/2012/929650

**Published:** 2012-04-19

**Authors:** Priyanka Verma, Ajay Kumar Mathur, Sheetal Prasad Jain, Archana Mathur

**Affiliations:** ^1^Division of Plant Biotechnology, Central Institute of Medicinal and Aromatic Plants (CIMAP), Council of Scientific and Industrial Research, PO CIMAP, Lucknow 226015, India; ^2^Department of Botany, Central Institute of Medicinal and Aromatic Plants (CIMAP), Council of Scientific and Industrial Research, PO CIMAP, Lucknow 226015, India

## Abstract

Twenty-three pharmaceutically important plants, namely, *Elaeocarpus spharicus*, *Rheum emodi*, *Indigofera tinctoria*, *Picrorrhiza kurroa*, *Bergenia ciliata*, *Lavandula officinalis*, *Valeriana wallichii*, *Coleus forskohlii*, *Gentiana kurroo*, *Saussurea lappa*, *Stevia rebaudiana*, *Acorus calamus*, *Pyrethrum cinerariaefolium*, *Aloe vera*, *Bacopa monnieri*, *Salvia sclarea*, *Glycyrrhiza glabra*, *Swertia cordata*, *Psoralea corylifolia*, *Jurinea mollis*, *Ocimum sanctum*, *Paris polyphylla*, and *Papaver somniferum*, which are at the verge of being endangered due to their overexploitation and collection from the wild, were successfully established *in vitro*. Collections were made from the different biodiversity zones of India including Western Himalaya, Northeast Himalaya, Gangetic plain, Western Ghats, Semiarid Zone, and Central Highlands. Aseptic cultures were raised at the morphogenic level of callus, suspension, axillary shoot, multiple shoot, and rooted plants. Synseeds were also produced from highly proliferating shoot cultures of *Bacopa monnieri*, *Glycyrrhiza glabra*, *Stevia rebaudiana*, *Valeriana wallichii*, *Gentiana kurroo*, *Lavandula officinalis*, and *Papaver somniferum*. *In vitro* flowering was observed in *Papaver somniferum*, *Psoralea corylifolia*, and *Ocimum sanctum* shoots cultures. Out of 23 plants, 18 plants were successfully hardened under glasshouse conditions.

## 1. Introduction

Numerous drugs or their precursors used in the current pharmacopoeias originate from plant sources. Natural products or natural product-derived drugs comprise nearly 28% of all the new chemical entities launched into the market in the last 20 years [1]. Medicinal plant-based drugs have the added advantage of being simple, effective, and offering a broad spectrum of activity with well-documented prophylactic or curative actions. Medicinal plant products have also proved useful in minimizing the adverse side effects of various chemotherapeutic agents [2, 3]. The World Health Organization (WHO) has estimated that the present demand for medicinal plants is approximately US $14 billion per year. The demand for medicinal plant-based raw materials is growing at the rate of 15 to 25% annually, and according to an estimate of WHO, the demand for medicinal plants is likely to increase more than US $5 trillion in 2050. In India, the medicinal plant-related trade is estimated to be approximately US $1 billion per year [4]. Medicinal plants are living resource, exhaustible if overused and sustainable if used with care and wisdom. At present 95% collection of medicinal plant is from the wild. Current practices of harvesting are unsustainable, and many studies have highlighted depletion of resource base. Medicinal plants-based industries although old and vast are still being managed on traditional ethos and practices and lack a proactive and socially responsible image. Many studies have confirmed that pharmaceutical companies are also responsible for inefficient, imperfect, informal, and opportunistic marketing of medicinal plants. As a result, the raw-material supply situation is shaky, unsustainable, and exploitative. There is a vast, secretive, and largely unregulated trade in medicinal plants, mainly from the wild which continue to grow dramatically in the absence of serious policy attention with environmental planning. Confusion also exists in the identification of plant materials where the origin of a particular drug is assigned to more than one plant, sometimes having vastly different morphological and taxonomical characters; therefore, adulteration is common in such cases [5–7].

India is rich in medicinal plant diversity. All known types of agroclimatic, ecologic, and edaphic conditions are met within India [8]. The biogeographic position of India is so unique that all known types of ecosystems range from coldest place like the Nubra Valley with −57°C, dry cold deserts of Ladakh, temperate and Alpine and subtropical regions of the North-West and trans-Himalayas, rain forests with the world's highest rainfall in Cherrapunji in Meghalaya, wet evergreen humid tropics of Western Ghats, arid and semiarid conditions of Peninsular India, dry desert conditions of Rajasthan and Gujarat to the tidal mangroves of the Sunderban. India is rich in all the three levels of biodiversity—such as species diversity, genetic diversity, and habitat diversity [8]. There are about 426 biomes representing different habitat diversities that gave rise to one of the richest centres in the world for plant genetic resources [9]. Concerning the total number of flowering plant species, although only 18,665, the intraspecific variability found in them make it one of the highest in the world. Out of 18,665 plants, the classic systems of medicines like Ayurveda, Siddha, and Unani make use of only about 3000 plants in various formulations [7].

The continued growth of human populations and of *per capita* consumption has resulted in unsustainable exploitation of Earth's biological diversity, exacerbated by climate change, ocean acidification, and other anthropogenic environmental impacts [10]. Conservation of medicinal plants can be accomplished by the *ex situ*, that is, outside natural habitat by cultivating and maintaining plants through long-term preservation of plant propagules in plant tissue culture repositories [10]. Although species conservation is achieved most effectively through the management of wild populations and natural habitats (*in situ* conservation), *ex situ* techniques can be used to complement *in situ* methods and, in some instances, may be the only option for some species [11–13].

*In vitro *approaches for the conservation and the use of plant germplasm can offer some distinct advantage over alternative strategies. Some of these are as follows: (1) collection may occur at anytime independent of flowering period for each species (this assumes that seed material is not required), (2) there is the potential of virus elimination from contaminated tissue through meristem culture, (3) clonal material can be produced where this is useful for the maintenance of elite genotypes, (4) rapid multiplication may occur at any time where stocks are required using micropropagation procedures, (5) germination of difficult or immature seed or embryo may be facilitated for breeding programmes, and (6) distribution across the border may be safer, in terms of germplasm health status using *in vitro *cultures. Some more general positive advantages of *in vitro* techniques include the fact that storage space requirements are vastly reduced compared with field storage. Storage facilities may be established at any geographical location and cultures are not subject to environmental disturbances such as temperature fluctuation, cyclones, insect, pests, and pathogen [14, 15].

The present investigation deals with collection and *in vitro* conservation of twenty-three overexploited medicinal plants belonging to different biodiversity zones of Indian subcontinent, at various morphogenic levels and their glasshouse hardening. These plants are at the verge of being endangered, so the work presented here will be beneficial for the biological conservation as well as for the worldwide pharmaceutical industry.

## 2. Methods

### 2.1. Collection of Plant Material

**Figure 1:**
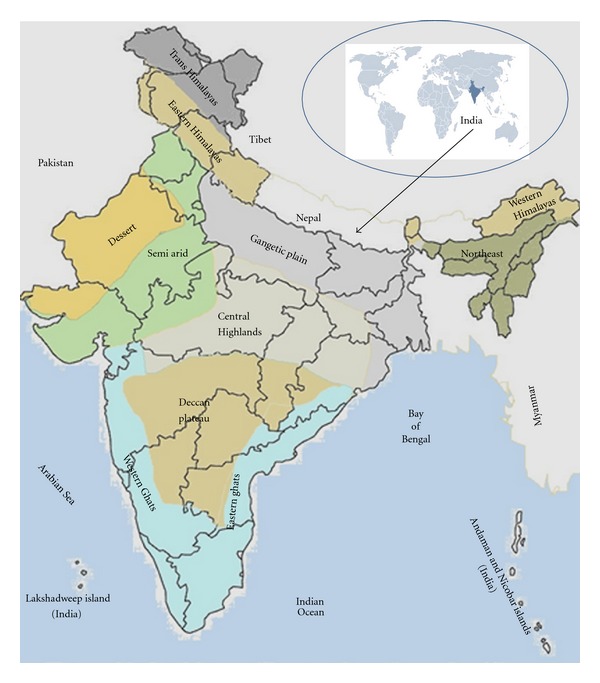
Different biodiversity zones of Indian subcontinent from where 23 plant species were collected.

The plant material of twenty-three medicinal plants [16], namely, (1) *Elaeocarpus spharicus*, (2)* Rheum emodi*, (3)* Indigofera tinctoria*, (4)* Picrorhiza kurroa*, (5)* Bergenia ciliata*, (6)* Lavandula officinalis*, (7)* Valeriana wallichii*, (8)* Coleus forskohlii*, (9) *Gentiana kurroo*, (10) *Saussurea lappa*, (11)* Stevia rebaudiana*, (12) *Acorus calamus*, (13) *Pyrethrum cinerariaefolium*, (14)* Aloe vera*, (15)* Bacopa monnieri*, (16)* Salvia sclarea*, (17)* Glycyrrhiza glabra*, (18)* Swertia cordata*, (19)* Psoralea corylifolia*, (20)* Jurinea mollis*, (21)* Ocimum sanctum*, (22)* Paris polyphylla*, and (23)* Papaver somniferum, *were collected from different biodiversity zones of India (Figure 1; Table 1). Collections were made in terms of different plant part like rhizome, tuber, leafy shoot, proliferating bud, rooted plant, seeds, and so forth.

### 2.2. Raising Aseptic Cultures and Multiplication

Various explants, namely, leaf, node, bulb, basal disc, and seeds, were first washed thoroughly for 2-3 hours under tap water (seeds left overnight for soaking). These were then washed with a detergent (cetrimide) which removes dust particles and clears the surface of the explant by removing hairs. Washed material was then treated with (4%) savlon for 2-3 minutes, followed by exposure to absolute alcohol for 30 seconds, and finally subjected to surface sterilization by 0.1% mercuric chloride. Treatment time varied from plant to plant and from explant to explant. While underground parts and basal discs were treated for 8–10 min, seeds, leaves, and nodes were treated for 1-2, 2-3, and 4-5 min, respectively. The explants were then thoroughly washed with sterile water (3-4 times). The explants were cut and inoculated on different media combinations.

More than 100 media combinations were tested for raising aseptic cultures of all the 23 plant species. According to the morphogenetic response required, medium recipes were made by using basal media [17] fortified with different ranges of 2,4-diphenoxyacetic acid (2,4-D), 2,isopentenyl adenine (2IP), ascorbic acid (AA), 6-benzylamino purine (BAP), casein hydrolysate (CH), indole-3-acetic acid (IAA), indole-3-butyric acid (IBA), kinetin (KN), and *α* naphthalene acetic acid (NAA). Cultures were incubated at 25 ± 2°C under 8 hours/16 hours photoperiod light 2500–3000 lux intensity and 75–85% relative humidity.

### 2.3. Synseed Production

The nodal segments and shoot tips of highly proliferating shoot cultures of different plant species were dissected from *in vitro* raised mother plant and used as explants for the synseed production. For encapsulation explants dispersed in 4% sodium alginate solution were suspended dropwise in 75 mM calcium chloride solution so that each drop contained single explant. The complexation time was 1 hr, and then the synseeds were thoroughly washed with sterile water and plated on MS basal medium for germination.

### 2.4. Glasshouse Hardening

Before transplantation, shoots were shifted to rooting medium. To establish root proliferation, actively growing shoots were transferred to respective rooting media (Table 2). When adequate rooted shoots were obtained, the rooted plantlets (mostly 20–30 days old) were transferred to glasshouse conditions. For that plantlets were carefully taken out without injuring the roots, thoroughly washed with running tap water to remove the adhered agar by gentle shaking in water filled plastic tray, and planted in a mixture of sterilized soil and vermicompost (1 : 1) in earthen pots. Temperature and humidity was gradually lowered according to the native conditions of the plant.

## 3. Results

Twenty-three medicinally important plants were successfully established *in vitro* at various morphogenic levels. Details pertaining to optimised media combination and morphogenic levels are given in the Table 2.* P. kurroa*, *L. officinalis*, *C. forskohlii*, *S. rebaudiana*, *P. cinerariefolium*, *B. moneeiri*, *G. glabra*, *S. cordata*, and *P. somniferum *showed both induction and proliferation of callus as well as shoots. *E. spehericus*, *J. mollis*, and *S. lappa* were established only at the callus level, while *I. tinctoria*, *B. ciliata*, *V. wallichii*, *G. kurroo*, *A. calamus*, *A. vera*, *S. sclarea*, *P. corylifolia*, *O. sanctum*, *P. polyphylla*, and *R. emodii *were maintained only at shoot level (Figure 2). All the shoot cultures showed first bud break within 20–30 days, but *P. polyphylla* bulb took longest time of six months to show first bud emergence. Out of these cultures, friable callus of only *G. glabra*, *L. officinalis*, and *P. somniferum *were able to give rise to fine suspensions. Cultures of *B. ciliata, P. somniferum, *and* J. mollis* showed excessive leaching of phenolics in the medium; therefore, they were subcultured frequently. 40–45-day old shoot cultures of *P. somniferum, P. corylifolia, *and* O. sanctum *were shown in *in vitro* flowering in 100%, 40%, and 70% cultures, respectively (Figure 2). In *P. Somniferum,* terminal white flowers were obtained which were either pentamerous or tetramerous. The five-lobed persistent stigma was prominent. Yellow coloured anthers were elliptical, ovoid, and numerous. Characteristic unilocular capsule formation was observed within 15 days of flower initiation. Similarly in *P. corylifolia *capitulate cylindrical inflorescence with white purplish flowers having unequal corolla, inserted stamens, and feathered stigma was observed. In case of *O. sanctum,* pentamerous flowers with white petals, four stamens surrounded single style, arranged in verticillaster manner were obtained. There were six flowers in each whorl. The shoots of *B. moneeiri*, *G. glabra*, *S. rebaudiana*, *V. wallichii*, *G. kurroo*, *L. officinalis*, and *P. somniferum* were highly proliferating; therefore, nodal segments of above plants were easily encapsulated to produce synthetic seeds. *B. moneeiri*, *S. rebaudiana*, *V. wallichii*, *L. officinalis*, *and P. somniferum* showed 100% germination rate of synseeds, while only 50% and 70% germination was observed in *G. glabra *and* G. kurroo*, respectively. Rooting was observed in all the plant species except *R. emodii* and *P. polyphylla*. Rooted plants were successfully transferred to the glasshouse conditions with a success rate of 30–60% (*B. ciliata*, *P. kurroa*, *I. tinctoria*, *G. kurroo*, *L. officinalis*, *S. cordata*, *A. calamus*, and* P. cinerariefolium,*), 70–90% (*C. forskohlii*, *P. somniferum*, *G. glabra*, *A. vera*, and* P. corylifolia*), and 100% (*B. moneeiri*, *O. sanctum*, *S. sclarea*, *S. rebaudiana*, and* V. wallichii*).

## 4. Discussion

India has been in focus for its high biodiversity, and this region has been a priority for leading conservation agencies of the world. World Wildlife Fund (WWF) has identified the entire Eastern Himalayas as a priority Global 200 Ecoregion while Conservation International has up scaled the Eastern Himalaya Hotspot [4, 7]. The region is rich in medicinal plants and many other rare and endangered taxa. Its high endemism in higher plants has qualified it to be a biodiversity “hotspot” [18]. Presently biological diversity of India is facing various threats like deforestation, degradation, agriculture, commercial plantations, and replacement of indigenous species with exotics [10]. Encroachment of forestland is a serious threat to forests and its conservation. It is estimated that 60% of the domestic herbivore population graze in the forest. The grazing causes soil compaction and heavy damage to the forest plantations and natural regeneration. Forest fires are also common and frequent affecting about 20% of the total forest area. *Ex situ* conservation of plants through *in vitro* technique is an ideal approach to combat above threats and restore biodiversity. *In vitro* techniques have been found to be useful in the propagation of large number of threatened species [11]. The first constrain for *in vitro* culture is the contamination which can be exogenous or endogenous. Exogenous contamination can be removed by thorough detergent wash while contamination from the internal sources can be potentially serious as many plant species harbour endophytic bacteria and fungi. In our study such condition was encountered in *B. ciliata*, *P. kurroa*, *G. kurroo*, *S. cordata*, and *A. calamus* and was successfully overcome by the incorporation of antibiotics (Ampicillin/Chloramphenicol 100 mg/I) in the respective media composition. Another major obstacle found during the establishment of the cultures was medium browning and excessive leaching in case of *B. ciliata*, *P. somniferum*, and* J. mollis.* Supplementation of 40 mg/I ascorbic acid in the respective media of* B. ciliata*, *P. somniferum* was able to control the leeching and browning up to certain extent, but frequent subculturing was the only feasible option observed in all the three plant species. The flowering process is one of the critical events in the life of a plant. This process involves the switch from vegetative stage to reproductive stage of growth and is believed to be regulated by both internal and external factors [19]. *In vitro* flowering observed in *P. somniferum*, *P. corylifolia*, and* O. sanctum* was highly significant as it is considered to be a convenient tool to study specific aspects of flowering like floral initiation, floral organ development, and floral senescence. Encapsulation technique used in the present study is very promising for conservation purposes as the protection provided to the plant material by encapsulation could increase its resistance to dehydration and low temperature, thus opening new possibilities for medium-term storage [20]. Lastly, 18 plant species were successfully hardened under the glasshouse conditions. During the acclimatization of different plant species in the glasshouse, two different stages were observed. In the first stage, plants adapted to the new environment with slow shoot and root growth. This stage is comparatively longer, but time period varies with the species to species. In the later stage, plants showed fast growth phase which was almost equal in most of the species.

The present microcloning/micropropagation protocols of 23 important medicinal plants will provide backup of collected germplasm *in vitro* at various levels of morphogenesis to supplement field gene bank. This tissue culture interface provides genetic enhancement of collected germplasm in terms of disease clearance and clonal propagation.

## 5. Conclusion

Medicinal plants are under the threat of overexploitation and biodiversity depletion. There is urgent need of their *ex situ* conservation. Collection and *in vitro* conservation of 23 pharmaceutically important plants in the present study opens fresh avenues towards the conservation and resource management of the overexploited medicinal plants.

## Figures and Tables

**Figure 2:**

*In vitro* established cultures of 23 plant species. (a)* Elaeocarpus spehericus*, (b)* Rheum emodii*, (c)* Indigofera tinctoria*, (d)* Picrorrhiza kurroa*, (e)* Bergenia ciliata*, (f)* Lavandula officinalis*, (g)* Valeriana wallichii*, (h)* Coleus forskohlii*, (i) *Gentiana kurroo*, (j) *Saussurea lappa*, (k)* Stevia rebaudiana*, (l) *Acorus calamus*, (m) *Pyrethrum cinerariefolium*, (n)* Aloe vera*, (o)* Bacopa moneeiri*, (p)* Salvia sclarea*, (q)* Glycyrrhiza glabra*, (r)* Swertia cordata*, (s)* Psoralea corylifolia*, (t)* Jurinea mollis*, (u)* Ocimum sanctum*, (v)* Paris polyphylla*, (w)* Papaver somniferum*, and (x)-(y) synseeds of *Bacopa moneeiri*. Arrows showing *in vitro* flowering.

**Table 1:** Details of the plants collected, medicinal value and their site of collection for *in vitro* conservation.

S. no.	Plant species	Family	Part used	Medicinal value	Zone of collection	Coordinates/altitude of site of collection
(1)	*Acorus calamus*	Araceae	Rhizome	Antiasthmatic, antibronchitis, antidiarrhoea, used in dysentery, stomach ache, and constipation	Western Himalayas	29°54′36′′N 79°37′46′′E; 1,500 mt
(2)	*Aloe vera*	Aloaceae	Leaves	Purgative, liver tonic, and used against skin allergies	Central Highlands	21°54′N 83°24′E; 215 mt
(3)	*Bacopa moneeiri*	Scrophulariaceae	Whole plant	Diuretic, cardiac, nervine, and tonic, improve intellect, used against asthma, hoarseness, insanity, epilepsy, potent nervous tonic, and antianxiety agent	Western Ghats	10°31′N 76°13′E; 2.83 mt
(4)	*Bergenia ciliata*	Saxifragaceae	Whole plant	Used against pimples, sun burn, stomach ache, muscular pain, and running eyes	Western Himalaya	25°34′N 91°53′E; 1,525 mt
(5)	*Coleus forskohlii*	Labiateae	Roots	Antiglaucoma, antiplatelet, bronchospasmolytic, cardiotonic, hypotensive, antiaging, antiallergic, smooth muscle, and arterial relaxant, antiasthmatic	Western Himalaya	29°54′36′′N 79°37′46′′E; 1,500 mt
(6)	*Elaeocarpus spehericus*	Elaeocarpaceae	Fruits/seed kernel	Expectorant, used against nervous weakness, insanity, epilepsy, melancholia, mania, other mental disorders, insomnia, headache, jaundice, hypertension, and fever	Central Highlands	21°08′N 81°23′E; 298 mt
(7)	*Gentiana kurroo*	Gentianaceae	Roots	Anthelmintic, anti-inflammatory, antiseptic, bitter tonic, cholagogue, emmenagogue, febrifuge, refrigerant, stomachic	Northeast Himalaya	27°0′N 88°16′E; 5,307 mt
(8)	*Glycyrrhiza glabra *	Fabaceae	Roots/rhizomes	Expectorant, diuretic, mild laxative, antiarthritic, antiinflammatory, antibiotic, antiviral, antiulcer, memory stimulant, antitussive, aphrodisiac, antimitotic, estrogenic, antioxidant, anticaries agent, antineoplastic, anticholinergic, antidiuretic	Gangetic plain	26°51′N 80°55′E; 128 mt
(9)	*Indigofera tinctoria*	Fabaceae	Leaves	Blood purifier (detoxify) reduces inflammation and alleviate pain antiseptic, stimulant	Gangetic plain	26°51′N 80°55′E; 128 mt
(10)	*Jurinea mollis*	Compositae	Root	Eruptions, decoction is given in colic, puerperal fever	Western Himalaya	31°58′N 77°06′E; 1220 mt
(11)	*Lavandula officinalis*	Labiateae	Whole plant	Known for essence, stimulant, sternutatory and tonic	Semiarid Zone	30.83°N 76.93°E; 656 mt
(12)	*Ocimum sanctum*	Labiateae	Leaves/seeds	Diaphoteric, antiperiodic, stimulating, expectorant, and anticatarrhal	Gangetic plain	26°51′N 80°55′E; 128 mt
(13)	*Papaver somniferum*	Papaveraceae	Capsule	Narcotic, analgesic, muscle relaxant, and antimicrobial agent	Gangetic plain	26°51′N 80°55′E; 128 mt
(14)	*Paris polyphylla*	Trilliaceae	Roots	Analgesic, anthelmintic, antiphlogistic, antispasmodic, antitussive, narcotic	Western Himalaya	30°25′N 79°20′E; 2,500 mt
(15)	*Picrorrhiza kurroa*	Scrophulariaceae	Root/stem	Used in fever, gastralgia, as laxative, liver complaints, anaemia. and jaundice	Western Himalaya	29°49′23′′N 79°26′54′′E; 2,286 mt
(16)	*Pyrethrum cinerariefolium*	Compositae	Whole plant	Febrifuge, stimulatory, and stimulant of nervous system, insecticide	Western Himalaya	29°54′36′′N 79°37′46′′E; 1,500 mt
(17)	*Psoralea corylifolia*	Papilionaceae	Seeds	Against bacteria causing skin diseases, leucoderma, leprosy, and psoriasis	Western Ghats	12°58′N 77°34′E; 920 mt
(18)	*Rheum emodii*	Polygonaceae	Roots	Stomach pains, constipation, dysentery, swelling of throat, and tonsillitis	Western Himalaya	32°22′N 77°14′E;3,979 mt
(19)	*Salvia sclarea*	Labiateae	Leaves/flower tops	Antidepressant, anticonvulsive, antispasmodic, antiseptic, aphrodisiac, astringent, bactericidal, carminative, deodorant, digestive, emmenagogue, euphoric, hypotensive, nervine, sedative, stomachic, uterine and nerve tonic	Western Himalaya	31°58′N 77°06′E; 1,220 mt
(20)	*Saussurea lappa*	Compositae	Root	Antiseptic, disinfectant, bronchitis, asthma, flatulence, and cardiac complaints	Northeast Himalaya	27°0′N 88°16′E; 5,307 mt
(21)	*Swertia cordata*	Gentianaceae	Whole plant	Blood purifier, febrifuge, anthelmintic, antimalarial, antidiarrhoea	Western Himalaya	29°49′23′′N 79°26′54′′E; 2,286 mt
(22)	*Stevia rebaudiana*	Asteraceae	Leaves	Sweetener (regulate blood sugar), increase energy level, mental alertness, appetite, vasodilator, skin care	Western Ghats	10°31′N 76°13′E; 2.83 mt
(23)	*Valeriana wallichii*	Valerianaceae	Roots	Useful in disease of eye, blood, liver, and spleen enlargement. Also useful in cleaning voice, headache	Western Himalaya	30°27′N 78°05′E; 1,876 mt

**Table 2:** Morphogenic level along with the optimised medium and glasshouse hardening status of 23 plant species processed for *in vitro* conservation.

S. no.	Plant species	Explant used	Callus	Cultures established at the level of					
				Suspension	Axillary/multiple shoots	Flowering	Synseed	Rooted plants	Time taken/success rate during hardening
(1)	*Acorus calamus*	Node	−	−	+ 5.0KN*	−	−	+ 3.0IBA	3-4 wks/60%
(2)	*Aloe vera*	Basal disc	−	−	+ 5.0KN**	−	−	+ 1.0IAA	3-4 wks/90%
(3)	*Bacopa moneeiri*	Leaf	+ 3.0BAP; 1.0IAA	−	+ MS0***	−	+	+ MS0	2 wks/100%
(4)	*Berginia ciliata*	Node	−	−	+ 3.0BAP; 0.1IAA; 40AA	−	−	+ 3.0IAA	5-6 wks/30%
(5)	*Coleus forskohlii*	Node	+ 2.5NAA	−	+ 0.2BAP	−	−	+ 2.5IAA	3-4 wks/70%
(6)	*Elaeocarpus spehericus*	Leaf	+ 2.0 NAA; 0.5KN	−	−	−	−	−	−
(7)	*Gentiana kurroo*	Node	−	−	+ 5.0KN	−	+	+ 2.0IAA	5-6 wks/50%
(8)	*Glycyrrhiza glabra *	Node	+ 0.5NAA; 0.5-2,4-D; 1000CH	+ 0.5NAA; 0.5-2,4-D; 1000CH	+ 2.5KN	−	+	+ 1.0IAA	2-3 wks/80%
(9)	*Indigofera tinctoria*	Seeds	−	−	+ 1.0BAP	−	−	+ 0.1IAA	5-6 wks/40%
(10)	*Jurinea mollis*	Leaf	+ 3.0BAP; 1.0IAA)	−	−	−	−	−	−
(11)	*Lavandula officinalis*	Seeds	+ 0.5 NAA; 0.5-2,4-D; 0.5 KN	+ 0.5NAA; 0.5-2,4-D; 1000CH	+ 2.5KN; 0.1NAA	−	+	+ 0.5IAA	5-6 wks/50%
(12)	*Ocimum sanctum*	Node	−	−	+ 3.0BAP; 1.0IAA	+	−	+ 1.0IAA	3-4 wks/100%
(13)	*Papaver somniferum*	Seeds	+ 1.0-2,4-D; 0.1KN; 40AA	+ 2.0–2,4-D; 0.25KN	+ 0.5-2IP; 0.1 IAA	+	+	+ 1.0IAA	5-6 wks/70%
(14)	*Paris polyphylla*	Bulb	−	−	+ 2.0BAP	−	−	−	−
(15)	*Picrorrhiza kurroa*	Leaf/node	+ 1.0-2,4-D; 0.5BAP	−	+ 5.0KN	−	−	+ 3.0IBA	5-6 wks/40%
(16)	*Pyrethrum cinerariefolium*	Leaf/node	+ 2.0-2,4-D; 1.0KN	−	+ 5.0KN; 2.0BAP; 1.0IBA	−	−	+ 1.0IBA	5-6 wks/60%
(17)	*Psoralea corylifolia*	Node	−	−	+ 5.0KN; 2.0BAP; 1.0IBA	+	−	+ 1.0IBA	3-4 wks/90%
(18)	*Rheum emodii*	Basal disc	−		+ 3.0BAP; 1.0IAA	−	−	−	−
(19)	*Salvia sclarea*	Node	−	−	+ 5.0KN; 2.0BAP; 1.0IBA	−	−	+ 1.0IBA	5-6 wks/100%
(20)	*Saussurea lappa*	Leaf	+ 1.0-2,4-D; 0.5BAP	−	−	−	−	−	−
(21)	*Swertia cordata*	Leaf/node	+ 2.0-2,4-D; 1.0KN	−	+ 5.0KN; 2.0BAP; 1.0IBA	−	−	+ 3.0IBA	5-6 wks/50%
(22)	*Stevia rebaudiana*	Leaf/node	+ 2.0-2,4-D; 0.25KN	−	+ 0.2BAP	−	+	+ 0.1IAA	4-5 wks/100%
(23)	*Valeriana wallichii*	Node	−	−	+ 2.5KN	−	+	+ MS0	3-4wks/100%

*All the medium recipes were formulated using Murashige and Skoog [17] basal medium; **concentration of all the used hormone is expressed in mg/I; ***MS0: MS basal without hormone supplementation.

2,4-D: 2,4-diphenoxyacetic acid; 2IP: 2,isopentenyl adenine; AA: ascorbic acid; BAP: 6-benzylamino purine; CH: casein hydrolysate; IAA: indole-3-acetic acid; IBA: indole-3-butyric acid; KN: kinetin; NAA: *α* naphthalene acetic acid; wks: weeks.
